# Microscopic colitis: Struggling with an invisible, disabling disease

**DOI:** 10.1111/jocn.14916

**Published:** 2019-05-29

**Authors:** Katarina Pihl Lesnovska, Andreas Münch, Henrik Hjortswang

**Affiliations:** ^1^ Department of Gastroenterology and Department of Clinical and Experimental Medicine Linköping University Linköping Sweden

**Keywords:** inflammatory bowel disease, microscopic colitis, qualitative method, quality of life

## Abstract

**Background and aims:**

Microscopic colitis causes chronic or recurrent nonbloody, watery diarrhoea, which is associated with urgency, faecal incontinence and abdominal pain. The patient's health‐related quality of life is often impaired. In microscopic colitis, health‐related quality of life has been studied using questionnaires originally constructed and validated for patients with inflammatory bowel disease. The aim of this study was to explore the impact of microscopic colitis on everyday life.

**Methods and results:**

Inductive, qualitative, semi‐structured interviews were performed with 15 persons suffering from microscopic colitis. Content analysis was used to explore the impact of the condition on everyday life. The study followed the consolidated criteria for reporting qualitative research. The qualitative inductive content analysis generated one theme and five subthemes. The theme was “struggling with an invisible, disabling disease.” The five subthemes were as follows: physical experience of bowel function; associated symptoms affecting quality of life; impact of the disease on everyday life; disease‐related worry; and strategies for managing everyday life.

**Conclusions:**

The semi‐structured interviews with persons suffering from microscopic colitis provided a wide spectrum of answers to the question of how everyday life is affected. Microscopic colitis can be a disabling life experience, and patients develop different strategies to adapt, cope and regain their previous performance level.

**Relevance to clinical practice:**

There are new and interesting findings in our study that everyday life still remains affected even when patients are in remission. These findings have relevance in clinical practice and may create a better understanding of the patient's symptoms and situation.

**What does this paper contribute to the wider global clinical community?**

- The struggle for microscopic colitis (MC) patients starts prior to diagnosis as the disease cannot be found with objective methods applied at the primary care facilities and is perceived to be “invisible”. Colonoscopy must be considered when seeing patients with frequent diarrhoeal and healthcare professionals must recognise the symptoms as early as possible in order to help patients by providing timely diagnosis and treatment.
- Worry is not a rational though. Several participants described disease‐related worry, which remained even when in clinical remission. Although serious complications in MC are rare, patients can still experience worry about, for example, colon cancer or the need for surgery. It is of great importance that healthcare professionals inform that the risk of such concerns becoming reality is very low in MC.

## 1 INTRODUCTION

Microscopic colitis (MC) is an inflammatory bowel disease (IBD), and MC is an overall term for two subtypes, collagenous colitis (CC) and lymphocytic colitis (LC). The diagnosis is based on typical histologic changes in the colorectal mucosa of patients with chronic diarrhoea. The incidence in the Western world of MC is increasing with a predominance in older women, but the aetiology of the disease remains unknown (Munch et al., 2012; Pardi, 2016). The most common symptoms are watery, nonbloody diarrhoea, severe urgency, faecal incontinence, abdominal pain and weight loss (Hjortswang et al., 2009). As there are still no reliable biomarkers for MC disease activity, the condition is defined by stool frequency and consistency (Hjortswang et al., 2009). The natural course of the disease is not associated with increased mortality, and severe disease‐related complications are rare, although the symptoms of active MC can severely impact health‐related quality of life (HRQoL), suggesting that HRQoL can be restored if remission is achieved (Hjortswang et al., 2011; Nyhlin, Wickbom, Montgomery, Tysk, & Bohr, 2014). Previous results show that HRQoL in MC patients with active disease is significantly impaired compared with both MC patients in remission and a general Swedish population (Hjortswang et al., 2011; Nyhlin et al., 2014).

## 2 BACKGROUND

The treatment goal is therefore to restore bowel function and thereby HRQoL. There is effective treatment available and the symptom burden can in most cases be alleviated with budesonide. However, the chronic inflammation tends to persist, and the symptoms generally return when the treatment is discontinued (Pardi, 2016). Urgency and the risk of faecal incontinence are linked to stool frequency and consistency, which improve as soon as the stool normalises (Hjortswang et al., 2009).

Although MC is not considered a life‐threatening condition, it is evident that it may profoundly impair HRQoL (Hjortswang et al., 2011). Previously, HRQoL in MC has been studied with questionnaires constructed and validated for patients with ulcerative colitis and Crohn's disease (Hjortswang et al., 2009). In order to investigate how patients with MC experience symptoms and how the symptoms impact on everyday life, an inductive qualitative study was performed, allowing patients to describe their experiences based on semi‐structured questions. This kind of study has to our knowledge not been published before, and therefore, we conducted inductive, qualitative, semi‐structured interviews with 15 patients suffering from MC.

## 3 AIM

The aim of the study was to explore the impact of microscopic colitis on everyday life.

## 4 METHODS

### 4.1 Method

Inductive, qualitative, semi‐structured interviews and problem‐driven content analysis were employed to explore the impact of MC on everyday life (Krippendorf, 2004). Content analysis is a research method for making replicable and valid inferences from data to their context, with the purpose of providing knowledge, new insights, a representation of facts and a practical guide to action (Krippendorf, 2004). The study followed the consolidated criteria for reporting qualitative research (COREQ; Appendix S1; Tong, Sainsbury, & Craig, 2007).

### 4.2 Participants and procedure

The data collection and analysis followed the COREQ checklist (Appendix S1). Participants were selected from an outpatient clinic in the south‐east of Sweden. The inclusion criteria were designed to ensure that the sample reflected the patient population and provided maximum variation in terms of age, sex, type of MC (CC or LC), disease activity and duration (Table 1). Those who agreed to participate signed a consent form. The first author (KPL) a female experienced IBD nurse and PhD contacted the participants to arrange a time and place for the interviews. All interviews were conducted by the first author in a conference room at the hospital. The interviews lasted for an average of 28 min (range 15–50 min) and were based on a semi‐structured interview guide (Table 2). In order to gain a deeper understanding of the phenomenon, probing questions were posed depending on the answers from the participants in order to encourage the participants to elaborate on and describe the impact of the disease on their everyday life (Tong et al., 2007).

**Table 1:** Demographic data

Variable	*N* = 15
Age mean (range)	62 (35–76)
Gender	
Female	11
Male	4
Place of birth	
Sweden	14
Other European country	1
Duration of disease in years, mean (range)	
Type of microscopic colitis (MC)	
Collagen colitis (CC)	11
Lymphocytic colitis (LC)	4
Symptom burden	
Remission	8
Recent relapse	6
Medical treatment	
No medical treatment	9
Loperamide	1
Budesonide	3
Anti‐TNF	2

**Table 2:** Interview guide

Questions
What physical and psychological symptoms do you associate with the active disease? Can you try to rank your symptoms regarding their degree of seriousness/impact on your everyday life? What is the most worrying aspect of the active disease? Do you have any bowel symptoms when the disease is not active? How do you manage the situation when the disease is active? Can you please tell me what the consequences of the active disease are for your life and how you are affected? What social consequences do you experience when the disease is active? What are your concerns?

### 4.3 Analysis

The interviews were digitally recorded and saved in a safe server. The interviews were transcribed verbatim by a professional secretary. The transcribed interviews were read on several occasions by the principal investigator to obtain an impression of the text as a whole. The analysis was performed through cut‐up paper transcript. Sentences relevant to the aim were identified and formed meaning units. The surrounding text was retained to preserve the context. The meaning units were condensed, after which they were compared to find similarities and differences. Similar meaning units were given the same code. The codes were then grouped into subthemes and a theme. The meaning units and codes were subsequently reread to determine their labels (Krippendorf, 2004). The coding was performed jointly by the research team.

### 4.4 Methodological considerations

Several steps were taken to ensure that the results are trustworthy and accurate. The semi‐structured interviews allowed the participants to explore the impact of MC on their everyday life and provide a more in‐depth insight into the phenomenon. The main theme and subthemes were represented in all interviews.

Inductive content analysis was chosen due to its suitability for exploring and describing experiences of a phenomenon that is not well explored. In this study, the in‐depth participant‐focused qualitative research approach allowed us to gather and analyse rich and detailed accounts of patients' perspectives on living with and adapting to MC.

The participants were selected in order to obtain the greatest possible variety of aspects of living with MC, which strengthens the validity of the results. In order to further strengthen validity, the first author conducted all the interviews to ensure that the participants focused on the same issues, as subtle changes in the wording of a question can lead to significant changes in the response. Validity was also strengthened by presenting relevant quotations to further illustrate the experience of living with MC.

### 4.5 Ethical considerations

The Regional Ethics Committee of Linköping approved the study (No. 2018/48‐31). In accordance with the Helsinki Declaration, the participants were assured of confidentiality throughout the process and informed about the voluntary nature of participation. The audio files and transcripts were numerically coded, and the way the data were analysed and presented ensured the participants confidentially.

## 5 RESULTS

**Figure 1:**
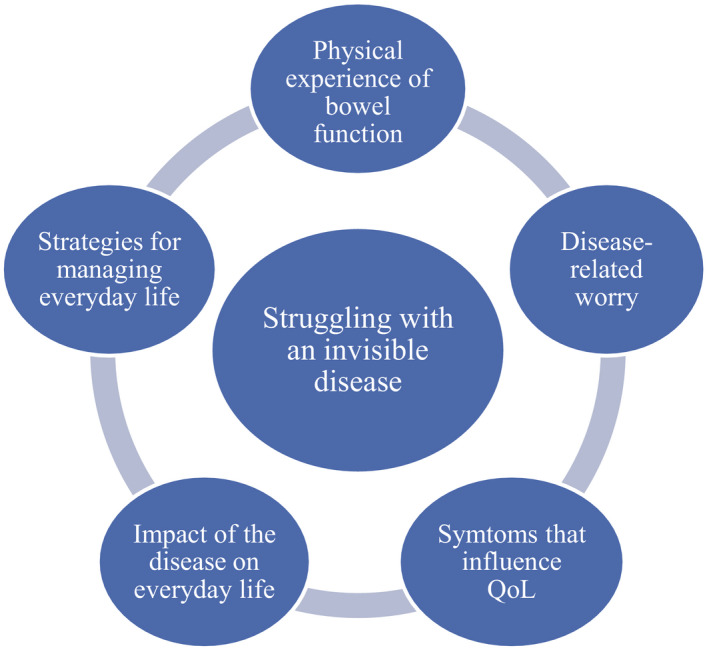
Themes and subthemes

Demographic details of the 15 participants are presented in Table 1. The qualitative inductive content analysis generated one theme and five subthemes (Figure 1). The theme was “struggling with an invisible, disabling disease”. The five subthemes were as follows: (a) physical experience of bowel function, (b) associated symptoms affecting QoL, (c) the impact of the disease on everyday life, (d) disease‐related worry and (e) strategies for managing everyday life.

### 5.1 Struggling with an invisible, disabling disease

The overall theme that emerged revealed that MC remains a histologic disease that appears invisible both when patients seek health care to obtain a diagnosis and when experiencing a lack of understanding from their social network. Several participants expressed that the time from seeking help from primary care to diagnosis was long, as no blood or faecal tests and no examinations reflected their own perception of the symptom burden. They also described struggling to make their next‐of‐kin and social network believe that they were ill when healthcare providers had difficulties explaining their complaints.

### 5.2 Physical experience of bowel function

The distressing symptoms of frequent watery stools and urgency, coupled with the fear of not reaching the toilet in time, were accompanied by embarrassment and taboo. The experience was unique to each individual and did not necessarily correlate with the amount or frequency of stools. Participants described a range of physical experiences and consequences of disturbed bowel function, including urgency, watery diarrhoea and faecal incontinence.

The worst is that when it comes it's so watery and that it happens so often. When it's really watery, very loose and occurs many times a day it's an ordeal! (BA‐15)

The worst is soiling yourself in public. (PS‐06)

Some also expressed that their bowel was much noisier and more bloated in the active phases of the disease. Several participants expressed that the smell of the faeces was different when having a flare compared to when they were in remission. The smell was described as chemical, similar to ammonia.

It's just water really and smells like some odd chemical! (PS‐06)

### 5.3 Associated symptoms affecting QoL

Some of the symptoms were directly associated with bowel function, such as abdominal pain, bloating, a feeling of satiety after meals and loss of appetite. These symptoms affected social activities, for example, eating alone due to poor appetite.

It's worse when I've eaten, I cannot eat large portions anymore. (UBJ‐10)

Other symptoms were not directly associated with MC but had appeared after or in connection with the diagnosis. These included joint pain, weight loss or gain, dry mouth, reduced ability to smell and taste as well as fatigue. The sudden reduction in the ability to taste and smell was described as a hindrance in a social context. Those who enjoyed preparing food stopped doing so because they were no longer able to determine what the food tasted like. That led to less social interaction and dinners with friends, which contributed to a feeling of sadness and loss of QoL. Fatigue was overwhelming and a common symptom among all the participants. Some also described the fatigue as a kind of weakness in the muscles when performing physical activities.

When it's at its worst I'm sort of washed out and drained. At that point I'm tired in a different way, it's hard to accomplish or do anything. (MK‐03)

I have a very bad sense of smell and taste. It disappeared when I became ill so cooking is difficult. (PS‐06)

Side effects from the treatment also contributed to anxiety and affected QoL.

### 5.4 The impact of the disease on everyday life

For some of the participants, the risk and fear of faecal incontinence led to isolation and withdrawal from social life and activities. Even those who did not totally withdraw from social activities pondered a great deal about what others thought about them in a social context. Things that others took for granted, such as attending a dinner with friends, became impossible during a flare.

If I'm invited somewhere I cannot accept because I know that I'll be unable to eat and then I might as well stay at home. If I eat something, I have to run to the toilet and that's no fun. (IN‐02)

One participant described wondering if people at work found it strange that she/he often ran to the toilet in the middle of meetings or coffee breaks.

At Liseberg [amusement park], if you stand in a queue for 1.5 hr and there is still an hour left to wait and you have to run to the toilet. Well then we have to queue again, as I cannot leave my child alone in the queue. It's very annoying for me but even worse when it affects someone else. (MK‐03)

Avoiding embarrassment caused by being unable to reach the toilet in time was a key theme. This very often restricted the participants in their everyday life. The participants had looked forward to pursuing activities when they retired, expecting to have more free time for social activities such as playing golf, riding a motorcycle and/or travelling but restrictions caused by the disease were a source of disappointment. On the other hand, participants who had retired expressed that the sense of control increased as they were less tied to schedules, which allowed them more flexibility to adapt to their bowel symptoms.

Yes, I've had to cancel one trip when my condition was at its worst because I felt that I would not manage to (pause) [I can't manage to fly if] I have to go to the toilet and there's a queue as I cannot cope with queues. (LNF‐12)

When the symptoms were bad, they experienced that it was impossible to take part in activities to the extent they wished. Some also expressed a feeling of being a burden to others when they had to cancel planned activities and were unable to fulfil their duties.

In the mornings I don't have much opportunity for activities, it happens that I cancel activities with friends; it depends on how I feel. (ME‐05)

Retired participants had been looking forward to taking part in activities with their grandchildren, baby‐sitting, helping their children and continuing to work to some extent, which was no longer possible.

Had things been different there's no doubt that I would have played golf and travelled to a greater extent and done more. I was active throughout my life and helped the children; I had a business here in the city. (BN‐08)

Fear of initiating an intimate relationship due to embarrassment and taboo around the disease and symptoms was also expressed. Their sex life was also affected as in addition to lacking libido they had a sense of being dirty or smelly.

Intimate life is affected. You do not feel as fresh as when you are in the middle of, you know… No but God now I have to go to the toilet we can hold up for a while. (BA‐15)

The participants also acknowledged that society expected that adults were able to control their bodily functions. They perceived that people in general had little or no understanding of the sudden need for a toilet. The constant need to know where to find a toilet reduces spontaneity and requires meticulous planning.

You have to mind what you're doing all the time; you dare not do anything spontaneously. (CO‐07)

Some expressed that it was easier to manage everyday life with strict routines rather than holidays and spontaneity.

### 5.5 Disease‐related worry

Living with MC creates worry and has a psychological impact. Even when in remission, an undercurrent of anxiety and worry related to bowel function remains.

I constantly have some form of worry, it's difficult to define what it's like but [I want to] overcome it and not allow it to take over my life… (AD‐04)

The most common worries were fear about developing cancer, undergoing surgery and the need for an ostomy bag. The fact that the disease is so unpredictable also contributed to a constant feeling of worry.

At times you are sitting at home and your thoughts wander, maybe it will develop into bowel cancer? Sometimes I have such thoughts. (IN‐02)

Several participants expressed anxiety about the future and coping with the worries experienced by next of kin.

I don't live fully, there's always a little fear. If I have planned something it's my stomach that decides, not me. (CE‐01)

The participants who were still working worried about how to tell their employer and co‐workers.

When I spoke to my colleagues after noticing that something was wrong, you know how people nod yet didn't take in what I was saying. I spoke to my boss but he just said aha, you have a little stomach problem! (PS‐06)

Although most of the participants tried to think in a positive way, the uncertainty related to their bowel function caused embarrassment and shame in many situations.

### 5.6 Strategies for managing everyday life

During the interviews, all participants described attempting to adapt to their condition. The strategies were situational avoidance such as not going out, avoiding certain places or planning the time, location and duration of activities. The strategies also included managing their symptoms, for example, emptying the bowel or taking loperamide before leaving home and avoiding food and/or alcohol intake.

I work as usual but I don't eat in the mornings, I only drink, in that way I don't need to go to the toilet and nothing will happen. (NH‐14)

Most participants ensured that they knew the location of the toilets and that they took incontinence products and/or extra clothing with them when leaving home.

The first thing I look for in a new place is the location of the toilet, other people look for emergency exits, and I look for toilets. (MK‐13)

One strategy was always carrying a box of matches, so that they could disguise the smell after a toilet visit. Another was learning to handle stressful situations, being open and telling others about the disease to make them aware of the situation.

I'm very open and talk a lot about it so that people will know. (AD‐04)

Some mentioned that they loved to travel but found it difficult to use certain kinds of public transport.

I have strategies, I travel to work by bus and my plan is to just get off and go behind some bushes and hope to catch the next bus. (BA‐15)

They adapted and opted to travel with, for example, a camper instead of an airplane. Another strategy for managing the disease was to find a seat near the aisle when at the theatre or cinema. Finally, the participants who informed their employer about suffering from MC related that their employer often provided practical support, mainly by allowing them to work flexible hours, thus making it possible for them to attend hospital appointments or deal with the impact of symptoms. The strategies were developed by the patients themselves, their next of kin or employer but usually without any input from healthcare professionals.

## 6 DISCUSSION

To the best of our knowledge, this is the first qualitative study that explores the impact of MC on everyday life as expressed by the patients themselves. Semi‐structured interviews provided patients with the opportunity to talk about their experiences to somebody outside their clinical team. This participant‐focused qualitative study provided rich and detailed information about patients’ experiences of living with and adapting to MC, in addition to revealing the physical, social and emotional impact of MC on everyday life.

It becomes apparent that the struggle experienced by MC patients starts prior to diagnosis, as the disease cannot be found with objective methods applied at primary care facilities and is perceived to be “invisible.” Patients have to cope with mistrust from healthcare providers and relatives, leading to frustration and disappointment. They are often obliged to wait a long time before receiving a referral for a colonoscopy and biopsy, which is the only way to make a diagnosis. The struggle of having an invisible disease underlines the importance of healthcare professionals being aware of the consequences of living with MC. All patients, especially older ones with chronic diarrhoea, should be referred for a colonoscopy, and biopsies should be obtained to confirm or rule out MC.

The findings reported here may help to inform clinical decision‐makers when addressing the large and currently unmet need for help with the bothersome symptoms experienced by people with MC. Frustration about having an invisible disease has also been highlighted by patients with ulcerative colitis and Crohn's disease (Purc‐Stephenson, Bowlby, & Qaqish, 2015). The participants in the present study reported that although they perceived themselves as sick, the fear that others would consider them lazy was both frustrating and exhausting. Such an experience could lead to lower self‐esteem or lack of self‐confidence (Purc‐Stephenson et al., 2015).

During a flare, the participants were focused on coping with the diarrhoea, urgency and risk of faecal incontinence. A great deal of thought and energy were spent on a daily basis to develop coping strategies that made embarrassing situations less likely. Patients with MC have to be constantly aware of the location of the nearest toilet. The worry about losing control over their bowel function, which is increased due to the watery stool consistency, has a major impact on their psychological well‐being. Their daily schedule and social contacts are arranged to take account of their bowel symptoms and plans are frequently cancelled, leaving them isolated with feelings of guilt and embarrassment. Their description of how the watery stools impacted on their everyday life was not always related to the frequency. This observation is important, as healthcare professionals often use bowel diaries and count stool frequency to evaluate the degree of disease, whereas stool consistency might have a greater impact on QoL.

The level of activity in each individual's life had a great impact on how the symptoms were subjectively perceived to hinder her/him in everyday life. This can also be seen in surveys of IBD patients. Lönnfors et al. (2014) showed that during a flare, 34% of the patients had to cancel or reschedule their plans frequently and 41% much of the time or sometimes. Even during remission, 37% still had to occasionally cancel or reschedule appointments due to IBD (Lonnfors et al., 2014). Other symptoms not related to the bowel also had an impact on everyday life. Among the main complaints was fatigue, which is one of the most common symptoms reported in Crohn's disease and ulcerative colitis (Czuber‐Dochan et al., 2014). It is important to acknowledge fatigue, as this may encourage patients to talk about and consider it as a legitimate problem.

The participants in the present study expressed that, in particular, faecal incontinence led to isolation and withdrawal from social life and activities. They also thought a great deal about what others might think of them in a social context, which can be time‐ and energy‐consuming. In individuals diagnosed with MC, there is a clear risk of lack of social participation leading to social isolation. They often feel restricted in terms of fulfilling their ambitions in life, while having to abandon the ideas and plans they had made for their retirement. The participants' perception that their own home is the only safe place and their strategy of avoiding public transportation limit social interaction. Humans are meant to connect with others and lack of such ties can have a negative impact on health. The impact of social relationships on health has been shown in numerous studies (Orth‐Gomer, Rosengren, & Wilhelmsen, 1993; Stringhini, Viswanathan, Gedeon, Paccaud, & Bovet, 2013; Yang, McClintock, Kozloski, & Li, 2013). The Berkman‐Syme Social Network Index (SNI), which measures the structural dimension of social relationships by quantifying the number of social ties and participation in social organisations, may be of importance for health and survival. Furthermore, social isolation in terms of the absence of social contacts and participation, rather than specific spheres of interaction, increases general susceptibility to disease and mortality (Stringhini et al., 2013). Research has also found that social isolation is significantly associated with specific disease aetiology, such as coronary heart disease (Orth‐Gomer et al., 1993). The findings in the present study highlight that many persons with MC isolate themselves and avoid social activities. It is vital to address this aspect when meeting these patients, especially in the light of the fact that social isolation has a major impact on HRQoL.

Moreover, the participants expressed that talking about bowel symptoms is still taboo in society. For patients who live alone, the symptoms can be associated with fear of entering into an intimate relationship. For those already in a relationship, their intimacy and sex life were affected due to lack of libido and a sense of being dirty or smelling bad. These aspects are important and must be addressed to a greater extent in health care. All participants stated that the issue of sexual life and intimacy was never raised by healthcare professionals. Patients with IBD rate sexual functioning and body image high compared to other concerns, which may have a negative influence on their HRQoL (Jedel, Hood, & Keshavarzian, 2015). Approximately 35%–58% of patients with IBD report impaired sexual functioning due to their diagnosis (Marín et al., 2013). The frequency of sexual dysfunction is higher in patients with IBD than in healthy controls (Marín et al., 2013). A better understanding of the role of sexual functioning and body image in HRQoL for patients with MC is needed.

Several participants described disease‐related worry, which remained even when in clinical remission, as the sudden onset of MC makes it unpredictable. Although serious complications in MC are rare, patients can still experience worry about, for example, colon cancer or the need for surgery. Patients' worries related to IBD and its consequences have been recognised (Stjernman, Tysk, Almer, Strom, & Hjortswang, 2010). The need for an ostomy bag or surgery, developing cancer and dying early seems to be among the main concerns of patients with IBD (Stjernman et al., 2010) As these worries are also reported by patients with MC, it is of great importance that healthcare professionals inform them that the risk of such concerns becoming reality is very low in MC.

Strategies to adapt to a lifelong disease were described. Retired patients have often accepted their situation to some extent, whereas younger patients struggle hard to cope with all the expectations from family and work. Psychological characteristics such as coping strategies to target the individual disease burden may impair or improve HRQoL. Coping may be broadly defined as “cognitive and behavioural efforts to manage specific external or internal demands (and conflicts between them) that are appraised as taxing or exceeding the resources of a person” (Moreno‐Jimenez, Lopez Blanco, Rodriguez‐Munoz, & Garrosa Hernandez, 2007). Healthcare professionals should have a more structured method of guiding patients with MC to develop coping strategies that help them to live an active everyday life.

## 7 CONCLUSION

This study reveals how MC impacts on every aspect of patients' lives; the interplay between physical symptoms; the consequences for everyday life; and how patients see themselves and their lives. It shows that MC can be a disruptive life experience and how patients employ adaptation strategies in an effort to live as normally as possible.

It is important that healthcare professionals recognise the symptoms as early as possible in order to help patients by providing timely diagnosis and treatment. Listening to and believing their narratives are essential, especially as MC is often perceived as being invisible. Although we know that MC is a benign disease, patients' worries must be addressed.

## 8 RELEVANCE TO CLINICAL PRACTICE AND CLINICAL IMPLICATIONS

There are new and interesting findings in our study that everyday life still remains affected even when patients are in remission. These findings have relevance in clinical practice and may create a better understanding of the patient's symptoms and situation. The struggle for MC patients starts prior to diagnosis as the disease cannot be found with objective methods applied at the primary care facilities and is perceived to be “invisible.” Patients must deal with mistrust from healthcare providers and next of kin leading to frustration and disappointment. Healthcare professionals in primary care must be vigilant for symptoms such as watery diarrhoeas in order to set diagnosis to avoid delay in starting treatment.

## CONTRIBUTIONS

Study design: KPL, HH and AM; data collection: KPL; and analysis and manuscript preparation: KPL, HH and AM.
